# NMR-Based Fragment Screen of the von Hippel-Lindau Elongin C&B Complex

**DOI:** 10.1021/acsmedchemlett.5c00316

**Published:** 2025-07-03

**Authors:** Kangsa Amporndanai, Jade M. Katinas, Ashima Chopra, Olumide Kayode, Anish K. Vadukoot, Alex G. Waterson, Stephen W. Fesik

**Affiliations:** † Department of Biochemistry, 12327 Vanderbilt University School of Medicine, Nashville, Tennessee 37232-0146, United States; ‡ Department of Pharmacology, 12327 Vanderbilt University School of Medicine, Nashville, Tennessee 37232-6600, United States; § Department of Chemistry, 5718 Vanderbilt University, Nashville, Tennessee 37235, United States

**Keywords:** VHL, E3 ligase, PROTAC, fragment-based screen, NMR screen

## Abstract

von Hippel-Lindau (VHL) is an E3 ligase that has been widely exploited for the development of PROTACs to induce degradation of disease-associated target proteins. Nearly all VHL-recruiting PROTACs contain a hydroxyproline moiety based on the endogenous peptide substrate that occupies the HIF1α-binding site of VHL. However, the development of orally bioavailable PROTACs with hydroxyproline-based VHL ligands remains a significant hurdle, due to both the hydroxyproline and the peptidic nature of the VHL ligand. Here, we describe an NMR-based fragment screen against the VHL-Elongin C-Elongin B (VCB) complex. Several hits were shown by X-ray crystallography to bind to the HIF1α active site in VHL of the VCB complex, which opens the possibility for the discovery of new nonhydroxyproline-based VHL ligands for use in VHL-recruiting PROTACs.

## Graphical abstract

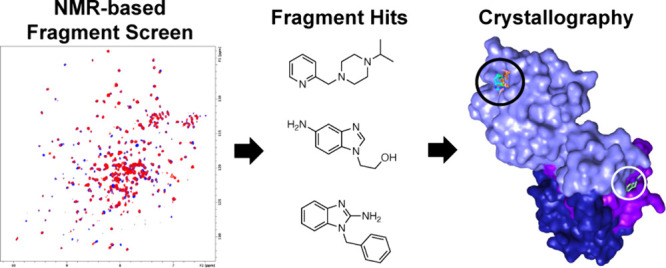

Targeted protein degradation (TPD) is a powerful therapeutic platform for eliminating disease-associated proteins via proteasomal degradation.^1^ Indeed, recent advancements in TPD address many targets that are historically considered undruggable using traditional drug discovery methods. Proteolysis Targeting Chimeras (PROTACs) utilize heterobifunctional small molecules that bind to a target protein and to an E3 ligase to promote ubiquitination of the target protein through induced proximity, resulting in subsequent destruction of the targeted protein by the proteasome.^2,3^

Although there are more than 600 human E3 ligases, only a handful of E3 ligases have been successfully used for TPD and only two E3 ligases, von Hippel-Lindau (VHL) and cereblon, are used in PROTACS with disclosed structures that have entered clinical trials.^4,5^ The high interest and applicability of VHL stems from its wide tissue expression profile and the availability of structurally characterized ligands to the protein.^6–8^ VHL serves as the substrate recognition domain of the E3 ligase complex consisting of Elongin B (Elo B), Elongin C (Elo C), cullin 2 (Cul2), and ring box protein 1 (Rbx1) (Figure 1).^9^ The VHL:EloB/C:Cul2:Rbx1 complex regulates turnover of hypoxia-inducible factor-1α (HIF1α) in response to changing oxygen level in cells. At adequate oxygen levels, HIF1α is hydroxylated at proline 564, allowing binding to VHL. This causes ubiquitination of HIF1α by the VHL:EloB/C:Cul2:Rbx1 complex and subsequent degradation by the proteosome. In hypoxic cells, proline 564 of HIF1α is not hydroxylated. VHL does not bind to nonhydroxylated HIF1α, and thus ubiquitination and degradation of HIF1α is not observed in hypoxic cells.^10^ The binding of the critical hydroxyproline VHL recognition feature has been structurally defined by the cocrystal structure of the VCB complexed to HIF1α peptide,^11^ and it has thus been used as a starting point to discover potent VHL ligands that incorporate chemical moieties N-terminal and C-terminal to the hydroxyproline.^12–15^

**Figure 1:**
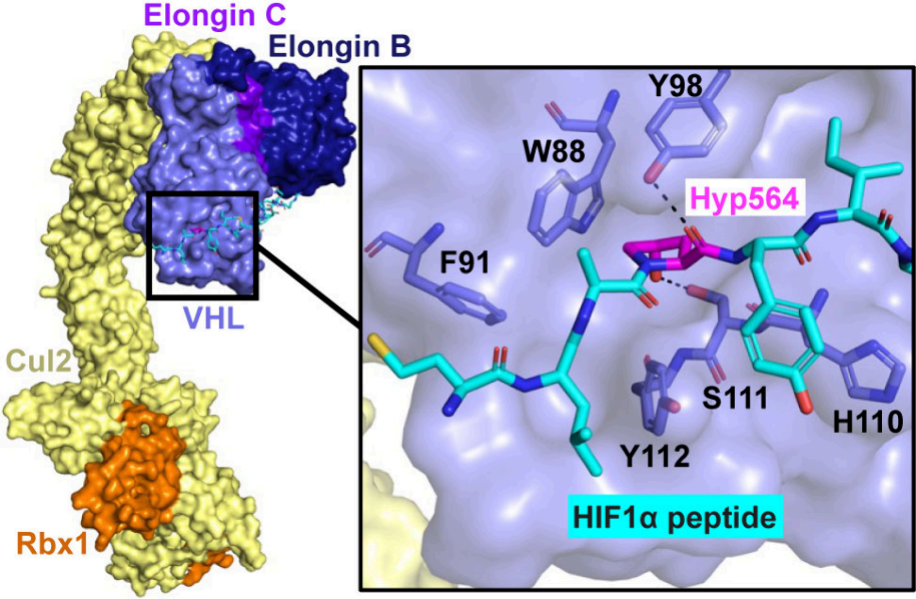
X-ray structure of the VHL:EloB/C:Cul2:Rbx1 complex (PDB: 5N4W) superimposed on the structure of VCB with the HIF1α peptide (cyan sticks, PDB: 1LM8). The structures show the key hydrogen bonds (black dashes) with hydroxyproline 564 (magenta sticks).

Indeed, over the years, many hydroxyproline-containing ligands for VHL have been designed and synthesized to achieve better affinity and physicochemical properties, partly to enable use in VHL-recruiting elements for PROTACS.^6,7,16^ VH032 was one of the first hydroxyproline-containing VHL ligands with nanomolar affinity *in vitro*, and has served as a template for the construction of the majority of VHL-recruiting PROTACs (Figure 2).^15^ Exploration of the utility of VHL ligands in PROTACs has progressed rapidly and now includes TPD of many oncology targets such as BRD4, KRAS, and SMARCA2, among others.^8,17–20^ To date, there are at least four VHL-recruiting PROTACs currently in clinical studies, including DT2216, KT-333, ASP3082, and PRT3789.^16^

**Figure 2:**
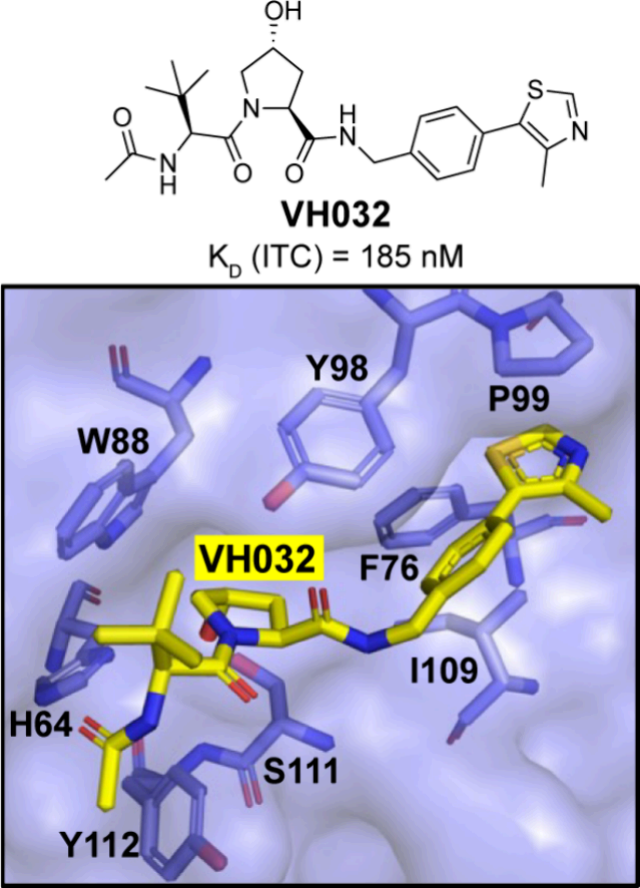
Chemical structure of VH032 and its cocrystallized structure with the VCB complex (yellow sticks, PDB: 4W9H).

A significant disadvantage of VHL-recruiting PROTACs is their generally limited oral bioavailability due to their poor physicochemical properties.^21–23^ Thus, new VHL ligands with improved properties are needed. To find new pharmacophores to improve upon or replace hydroxyproline-based VHL ligands, fragment-based approaches using a variety of biophysical techniques have been reported, including the use of isothermal titration calorimetry (ITC),^13,24^ fluorescence polarization assay (FPA),^12,13^ thermal shift assay (TSA),^13,24^ ligand-observed NMR,^12,13,24,25^ and screening covalent compounds using mass spectrometry.^26^ Although these studies have revealed ligandable pockets in VHL and Elongin C, none of the fragment hits from these studies has yet dethroned hydroxyproline-based ligands, which continue to dominate VHL-recruiting PROTAC development programs.^6^ Thus, the need for novel VHL ligands for use in the PROTAC field remains.

Fragment screening using protein-observed NMR is an ideal technique to utilize in the search for new chemical moieties that bind to VCB. Indeed, protein-observed NMR has successfully discovered fragment hits that have been subsequently elaborated to high affinity ligands for many undruggable target proteins.^27–30^

Here, we present the results from a fragment-based screen conducted by protein-observed NMR against uniformly ^15^N-labeled VCB. Fragment hits discovered in this screen unveil chemotypes that have not been previously observed to bind to the VCB complex. We also solved the X-ray cocrystal structures of VCB bound to fragment hits obtained from this screen. These results may serve as a useful starting point toward the discovery of novel small molecule ligands for the VCB complex and for developing VHL-based PROTACs with potentially better physicochemical properties than the hydroxyproline-based scaffolds currently in use.

As VCB is a large complex with a molecular weight of ∼42 kDa, the ^1^H–^15^N HSQC spectrum of the VCB complex recorded at 900 MHz (Figure 3A) contained broad peaks and overlapping resonances and was not ideal for conducting our fragment-based screen.^34^ In contrast, when relaxation-optimized spectroscopy (TROSY) was employed at 900 MHz (where the TROSY effect is optimized), a much better-resolved spectrum with narrower line widths was observed (Figure 3B),^31,32^ making it easier to analyze the chemical shift perturbations (CSPs) induced by fragment binding. Compared to others that have conducted fragment screens of the VCB complex, we utilized a much larger in-house fragment library containing a structurally diverse set of 13,824 compounds. To conduct the screen, we prepared large amounts of uniformly ^15^N labeled VCB and conducted a screen at a VCB concentration of 100 μM, using a mixture of 12 fragments at 800 μM of each fragment per screening sample. Fragment mixture hits were identified by monitoring CSPs in the ^1^H–^15^N TROSY spectrum in the presence of fragments compared to the spectrum acquired of ligand-free VCB. The specific fragments in each 12-fragment cocktail that were responsible for inducing the CSPs were identified by individually testing each fragment against VCB.

**Figure 3:**
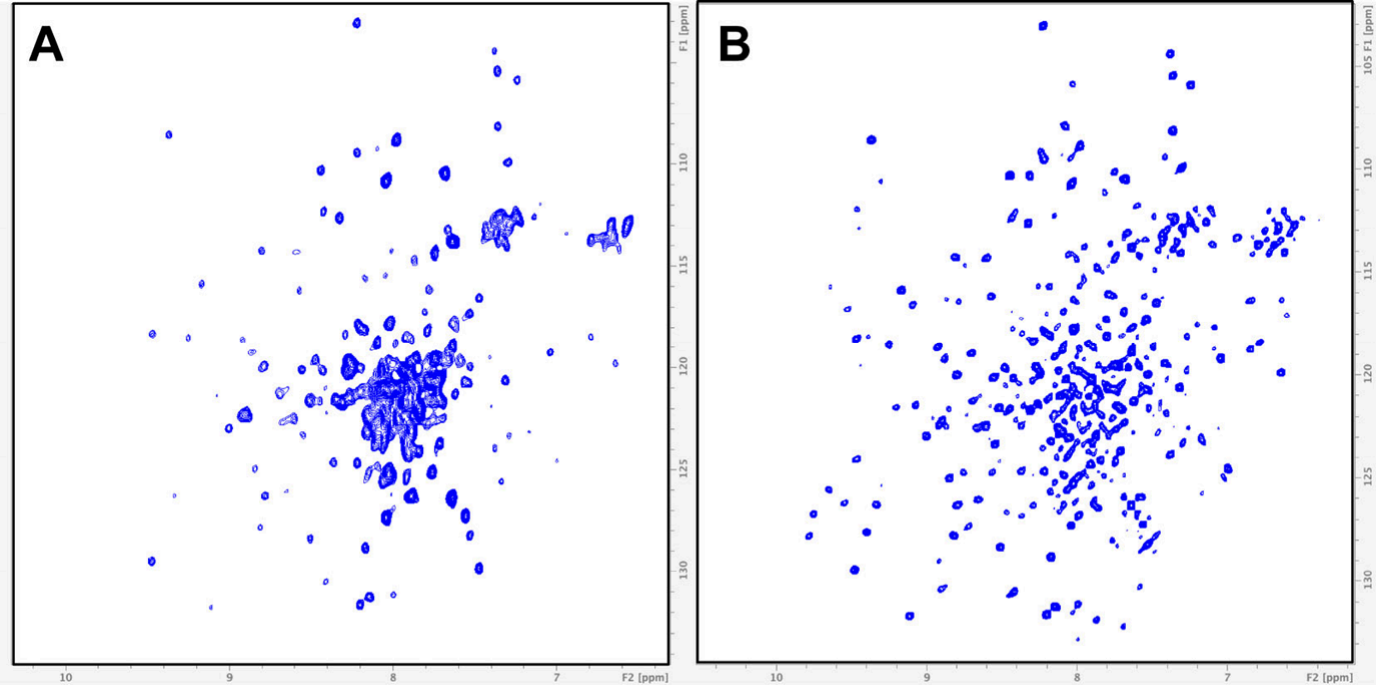
^1^H–^15^N (A) HSQC spectrum acquired at 900 MHz and (B) TROSY spectrum acquired at 900 MHz of the VCB complex.

Using this screening protocol, a total of 37 hits bound to VCB were identified (0.27% hit rate) with 6 sets of distinct patterns of resonance perturbations, suggesting that each set of fragments binds to different locations (Figure S1). The dissociation constants (K_D_s) of the fragment hits were determined by NMR titrations using a range of fragment concentrations from 0.31 mM to 10 mM. The chemical structures and binding constants of representative fragments of each set are shown in Figure 4A-D.

**Figure 4:**
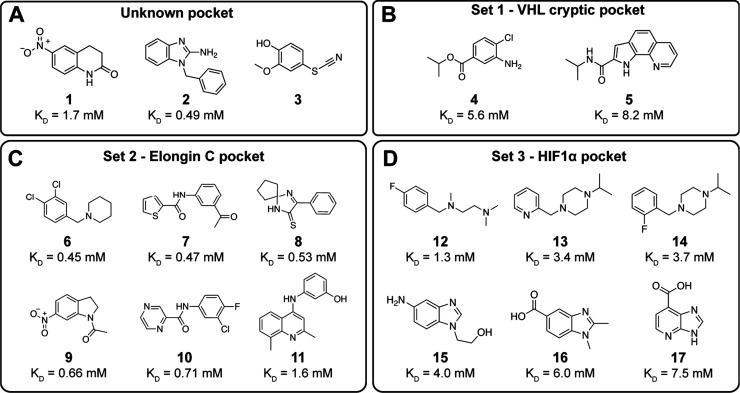
Chemical structures and NMR-derived binding affinities (K_D_s) of the fragment hits categorized by their binding pockets: (A) unknown pockets, (B) VHL cryptic pocket, (C) Elongin C pocket, and (D) HIF1α pocket.

To get a preliminary assessment of the binding location of the fragment hits, we analyzed the CSPs induced by previously identified VCB ligands^24^ (Figure S2) and compared these shifts to those observed for our ligands. Using this approach, we could map the likely binding pockets for 3 of the 6 sets of fragment hits. The fragment hits with different observed CSP patterns suggested that they bind to unknown pockets (Figure 4A).

Some of the CSPs of the newly identified hits show CSPs similar to those of MB756, which binds to a cryptic pocket in VHL (Set 1, Figure 4B).^24^ This pocket was previously determined by Ciulli’s group in a fragment-based screen by TSA and ligand-observed NMR,^24^ and experimentally validated by an X-ray cocrystallized structure.^24^ This pocket is located at the rear of VHL near the VHL:Cul2 interface (Figure S3). Although some residues in the cryptic pocket are involved in Chuvash polycythemia associated VHL mutants^33,34^ or substrate polyubiquitylation by E3 ligases,^35^ ligands to this pocket have not been widely exploited for making higher affinity VHL ligands or incorporated into PROTAC molecules.

A group of amides and heterocycles (Set 2, Figure 4C) showed an NMR shift pattern matching MB120 that was shown by X-ray crystallography to bind to a pocket on Elongin C (Figure S3).^24^ Several of these compounds showed <1 mM affinity to the Elongin C pocket. However, these hits are not suitable starting points for elaboration into improved VCB ligands for use in PROTACs because the pocket is located in the Cul2 interface of Elongin C. Ligand binding to this pocket would likely disrupt the necessary VHL and Cul2 complex formation and eventually interrupt the degradation machinery.

We next compared the chemical shifts induced by VH032, that bind to the HIF1α pocket, to those of our fragment hits and found several (Set 3, Figure 4D) that are predicted to bind to the HIF1α recognition site. These are the most interesting hits, since this site is a proven ligandable and PROTACable pocket on VHL. Chemotypes represented by this set of hits have not been reported to bind to VHL and do not contain a hydroxyproline. Thus, this set of fragments offers more attractive starting points for medicinal chemistry optimization to generate novel ligands in VHL-based PROTAC development.

To confirm the binding location of our fragment hits, we determined the X-ray structures of some of our hits bound to the VCB complex with a focus on those that bind to the HIF1α pocket. We solved four crystal structures of fragments bound to VCB by cocrystallization and soaking experiments (Figure 5A). Consistent with the NMR data, the Set 2 fragment hit **9** binds to the MB120 pocket within Elongin C. **9** is anchored by hydrogen bonds with E64 and E102 (Figure 5B), but as mentioned earlier, this compound is unsuitable for further elaboration.

**Figure 5:**
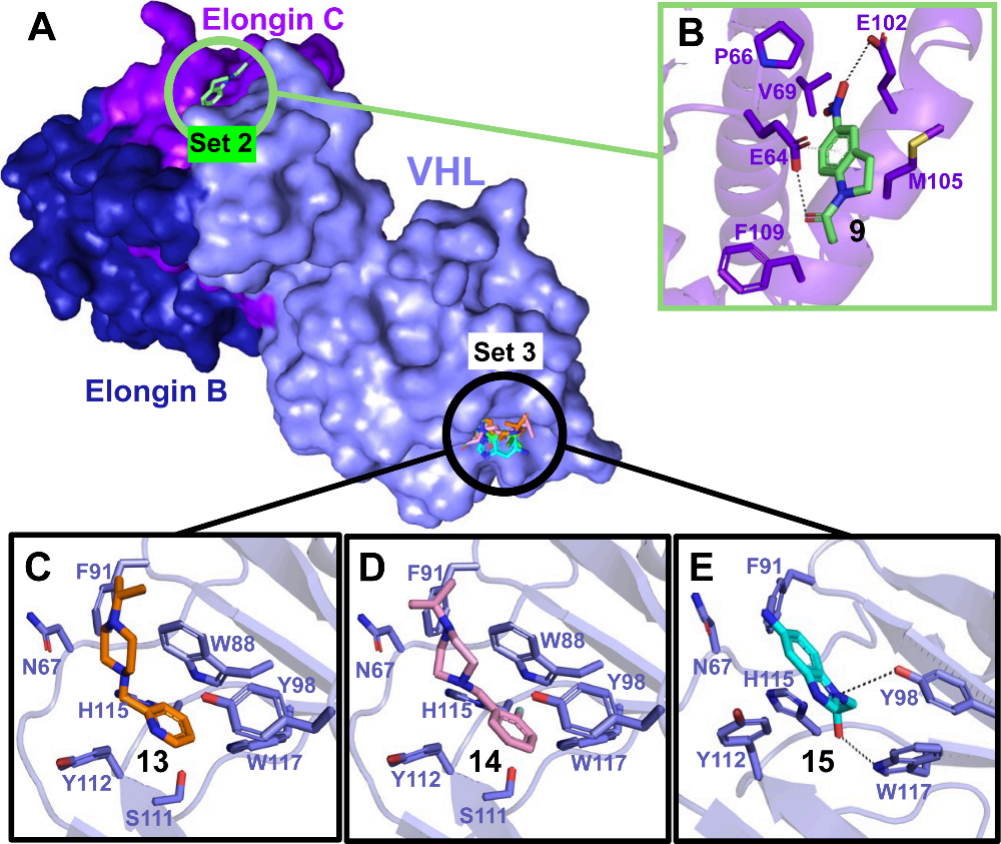
(A) Overlaid X-ray structures show the binding pockets of the Set 2 and Set 3 fragment hits within the VCB complex. Individual X-ray cocrystallized structures of (B) Set 2 fragment **9** (green sticks) that binds to Elongin C (purple sticks and cartoon) and Set 3 fragments (C) **13** (orange sticks), (D) **14** (pink sticks), and (E) **15** (cyan sticks) that bind to the HIF1α pocket of VHL (light purple sticks and cartoon).

The other 3 X-ray crystal structures were obtained with the Set 3 fragment hits that likely bind to the HIF1α recognition site, as evidenced by the NMR data. The X-ray structures of **13**, **14**, and **15** confirmed binding to VHL within the HIF1α active site (Figure 5C-E). Benzyl piperazines **13** and **14** are the most potent compounds among analogs of the same class and show similar binding poses within the HIF1α pocket (Figure 5C&D). These compounds each place an aromatic ring into the site defined by W88, Y112, H115, and W117. Notably, the piperazine group of **13** and **14** reaches out to the hydrophobic area near W88 and F91. We observed that the compound **15** engages in π–π stacking interactions within the binding site confined by the side chains of W88, Y112, H115, and W117 (Figure 5E). In addition, **15** can form two hydrogen bonds with Y98 and W117, likely explaining the higher affinity compared with other benzimidazole derivatives such as **16** and **17**.

We overlaid the X-ray structures of our fragment hits to the previously determined structure of VH032 bound to the VCB complex (PDB: 4W9H) (Figure 6A). We observe that our Set 3 fragments occupy a subpocket at the N-terminal end of the hydroxyproline core of VH032. Interestingly, all fragments bind to the pocket of *tert*-butyl group of VHL032 toward F91, but none of them bind to the pocket of acetyl amide group of VH032 which is commonly used as exit vector for connecting with a flexible linker in VHL-recruiting PROTAC design.^36^ To better visualize the subpockets within the HIF1α-binding site, the crystal structures of Set 3 fragments are individually shown in surface representation with the same perspective in Figure 6B-D. Based on the crystal structures, the benzimidazole fragment **15** and benzyl piperazine fragments **13** and **14** overlap with each other and with VH032 in the hydroxyproline subpocket. Fragments **13** and **14** occupy more hydrophobic area in the binding site and exhibit higher affinity than **15**. Together, this suggests the possibility of employing fragment merging to create VHL ligands with stronger affinity by merging or linking the fragment hits obtained in our screen together with the heavily optimized phenyl thiazole group of VH032 that binds to a pocket in VHL C-terminal to the hydroxyproline (Figure 6A).

**Figure 6:**
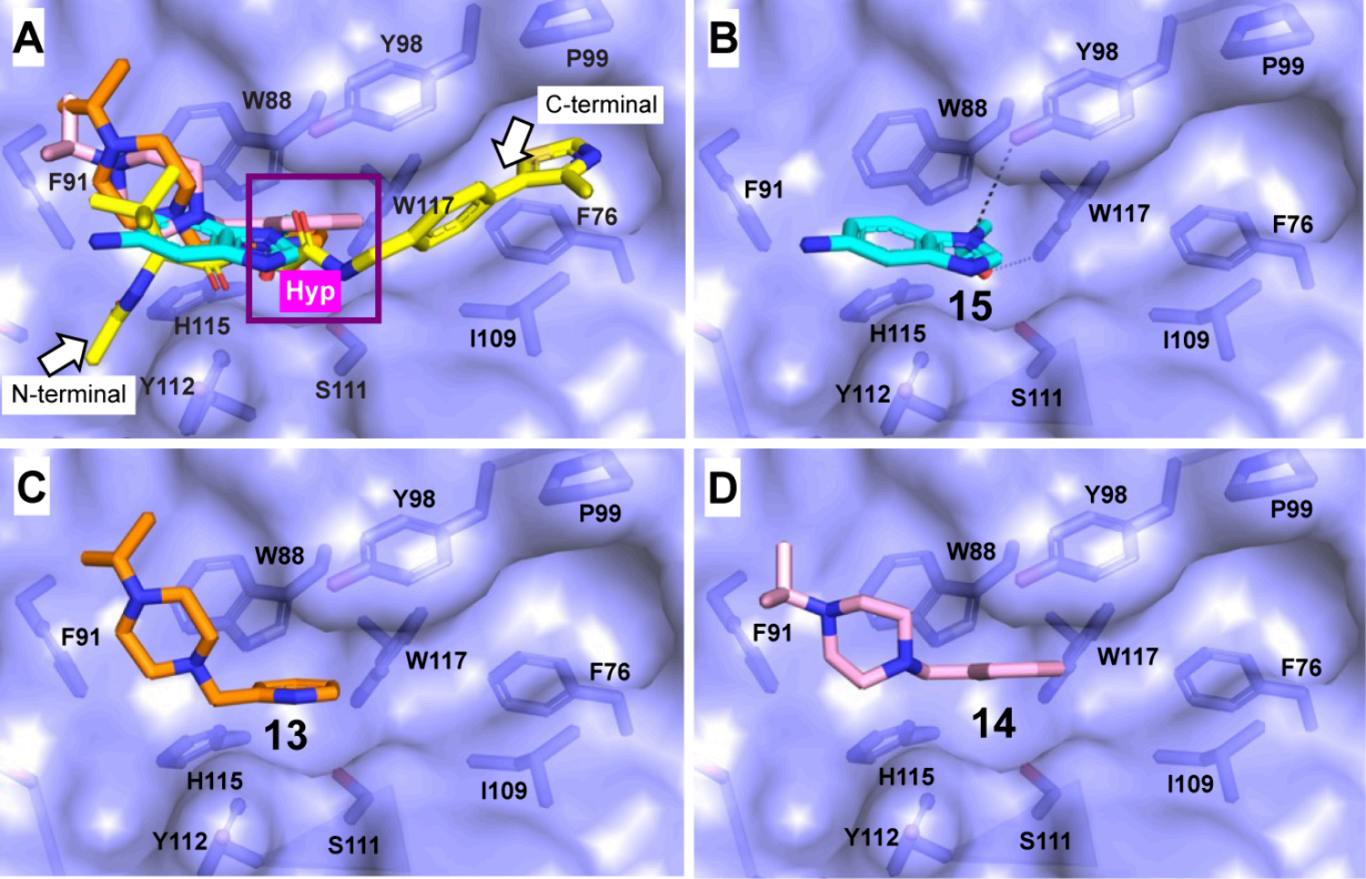
Surface representation of HIF1α-substrate binding site occupied by VH032 (PDB: 4W9H), and the Set 3 fragment hits. (A) overlaid structures of VH032 (yellow sticks), **15** (cyan sticks), **13** (orange sticks), and **14** (pink sticks). Individual structures of (B) **15**, (C) **13**, and (D) **14**. Hydroxyproline (Hyp) were highlighted in a purple box.

VHL is a well-established E3 ligase for PROTAC development, with molecules that recruit this ligase showing effective degradation of many target proteins. Although VHL ligands based on the hydroxyproline of the HIF1α substrate have been extensively optimized to achieve nanomolar affinity, PROTACs based on these ligands frequently fail to achieve good oral bioavailability, often limiting the advancement of VHL-recruiting degraders. To identify nonhydroxyproline starting templates for new VHL ligands, we conducted a protein-observed NMR-based fragment screen against ^15^N-labeled VCB complex and identified 37 fragment hits that bind to the complex. Co-crystal structures of 4 fragment hits bound to VCB were elucidated, revealing their binding modes to VHL. The fragment hits represent new chemotypes that bind the hydroxyproline subpocket within the HIF1α-recognition site and thus represent opportunities to redesign VHL ligands targeting the HIF1α pocket. Using the structural information, we propose that merging/linking these fragments together and to other pieces of known HIF1α binders might lead to new compounds with improved affinity for the HIF1α binding site of VHL. Such compounds may be useful for discovering new series of VHL-recruiting PROTACs with improved physiochemical properties and oral bioavailability, for the degradation of proteins to treat cancer and other diseases.

**Safety Statement**: No unexpected or unusually high safety hazards were encountered.

## Supplementary Material



## Notes

Supplementary Figures S1–S4, X-ray data collection and refinement statistics for fragments **9**, **13**, **14**, and **15**, experimental procedures of protein expression and purification, NMR experiments, and protein crystallography (PDF)

## Notes

Atom coordinates and structure factors for the VCB complex bound with fragments can be accessed in the Protein Data Bank via the following accession codes: 9OIM, fragment **9**; 9OIN, fragment **13**; 9OIO, fragment **14**; 9OIQ, fragment **15**. The authors will release the atomic coordinates upon article publication.

## ∥

Proteagen Biosciences Inc., Oakdale, Minnesota 55128, United States

## ABBREVIATIONS

BRD4: bromodomain-containing protein 4
CCR2: CC chemokine receptor 2
CSP: chemical shift perturbation
Cul2: cullin 2
K_D_: dissociation constant
K-Ras: Kirsten Rat sarcoma viral oncogene GTPase
HIF1α: hypoxia inducible factor 1α
HSQC: heteronuclear single quantum coherence
Hyp: hydroxy proline
ITC: isothermal titration calorimetry
PDB: protein data bank
PROTAC: proteolysis targeting chimera
Rbx1: ring box protein 1
SMARCA2: probable global transcription activator SNF2L2
SOFAST-HMBC: selective optimized flip angle short transient-heteronuclear multiple quantum coherence
TPD: target protein degradation
TROSY: transverse relaxation-optimized spectroscopy
TSA: thermal shift assay
VCB: VHL-Elongin C-Elongin B complex
VHL: von Hippel-Lindau protein
