# Supplementary material for: NMR-Based Fragment Screen of the von Hippel-Lindau Elongin C&B Complex
Source: ACS Med Chem Lett. 2025 Jul 3;16(8):1648–54. doi: 10.1021/acsmedchemlett.5c00316 (PMC12358976; doi:10.1021/acsmedchemlett.5c00316)
Supplement: Supplementary file 1 [file ml5c00316_si_001.pdf]

Supplementary Information

NMR-based Fragment Screen of the von Hippel-Lindau Elongin C&B complex

Kangsa Amporndanai[1], Jade M. Katinas[1], Ashima Chopra[1], Olumide Kayode[1†], Anish K. Vadukoot[1], Alex G. Waterson[2,3], Stephen W. Fesik [1,2,3*].

[1] Department of Biochemistry, Vanderbilt University School of Medicine, Nashville, Tennessee, 37232-0146, United States.

[2] Department of Pharmacology, Vanderbilt University School of Medicine, Nashville, Tennessee, 37232-6600, United States.

[3] Department of Chemistry, Vanderbilt University, Nashville, Tennessee, 37235, United States.

*Corresponding author

†Current address: Proteagen Biosciences Inc., Oakdale, Minnesota, 55128, United States.

Corresponding Author Contact

Stephen W. Fesik
Phone: +1 (615) 322-6303; Fax: +1 (615) 875-3236;
Email: Stephen.fesik@vanderbilt.edu

Table of Contents

Supplementary Figure S1-S4 2
X-ray data collection and refinement statistics for fragments **9**, **13**, **14**, and **15** 5
Experimental Procedures 6
    Protein Expression and Purification 6
    NMR Experiments 7
    Protein Crystallography 7

Supplementary Figures

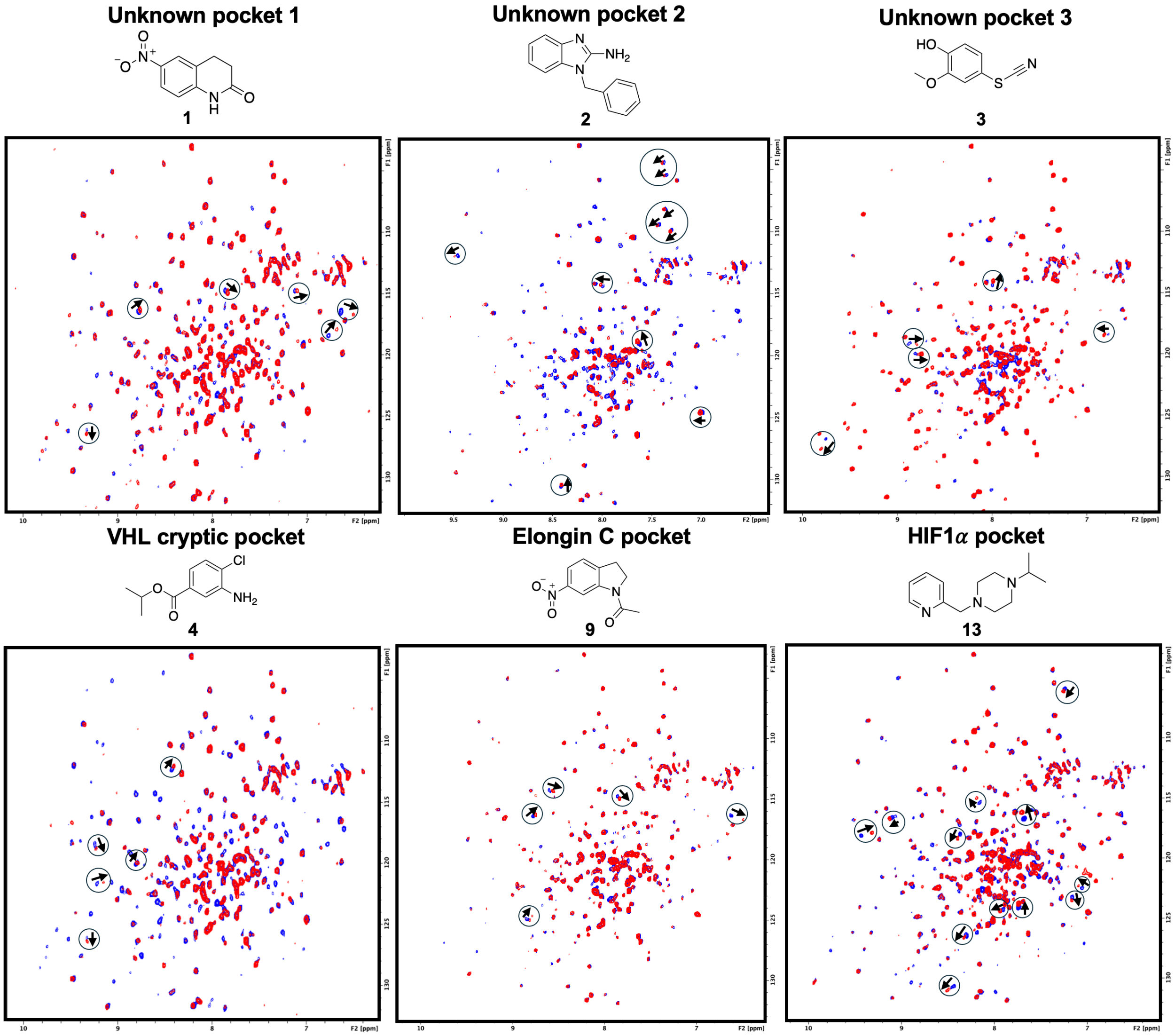

Figure S1. ^{1}H-^{15}N TROSY spectra of VCB without (blue) and with (red) fragments illustrate the different chemical shift patterns suggesting different binding pockets. Characteristic peaks of each class of fragment are highlighted in black circles with arrows.

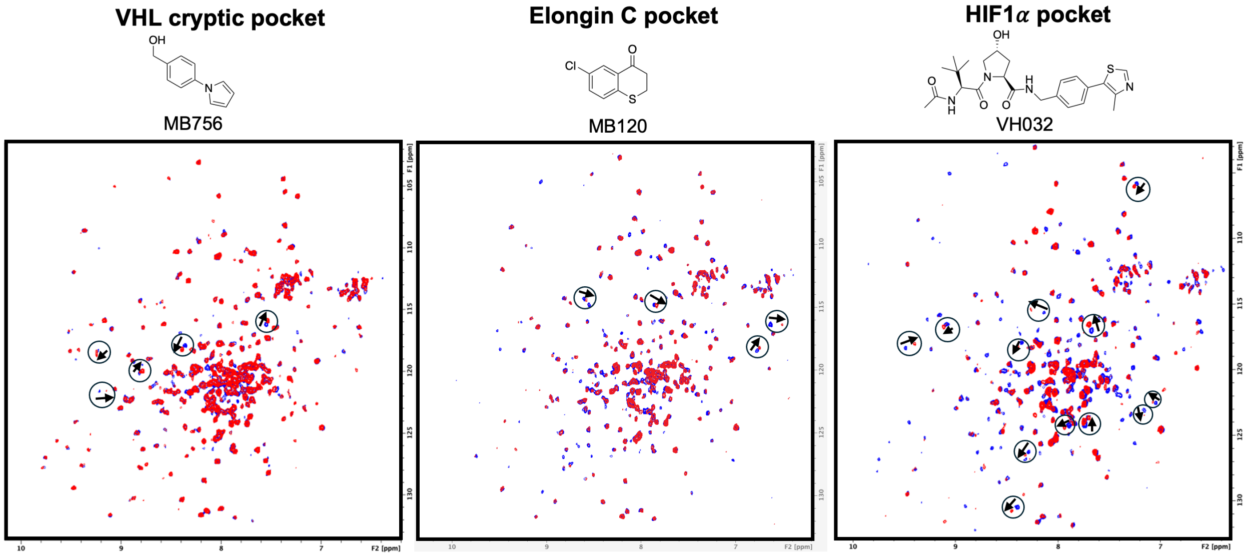

Figure S2. ^1H-^{15}N TROSY spectra of VCB without (blue) and with (red) compounds illustrate chemical shift patterns resulting from binding to specific known pockets. Characteristic peaks of each standard compounds are highlighted in black circles with arrows.

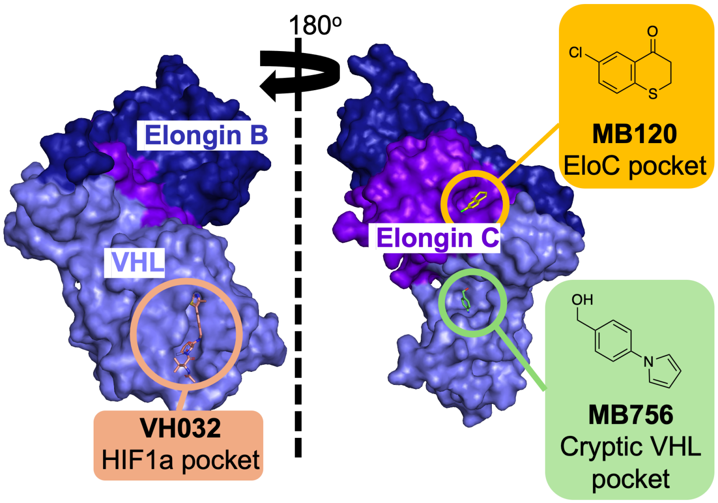

Figure S3. Overlaid crystal structures of MB756 (PDB: 6GMR), MB120 (PDB: 6GMX), and VH032 (PDB: 4W9H) show the binding pockets within the VCB complex.

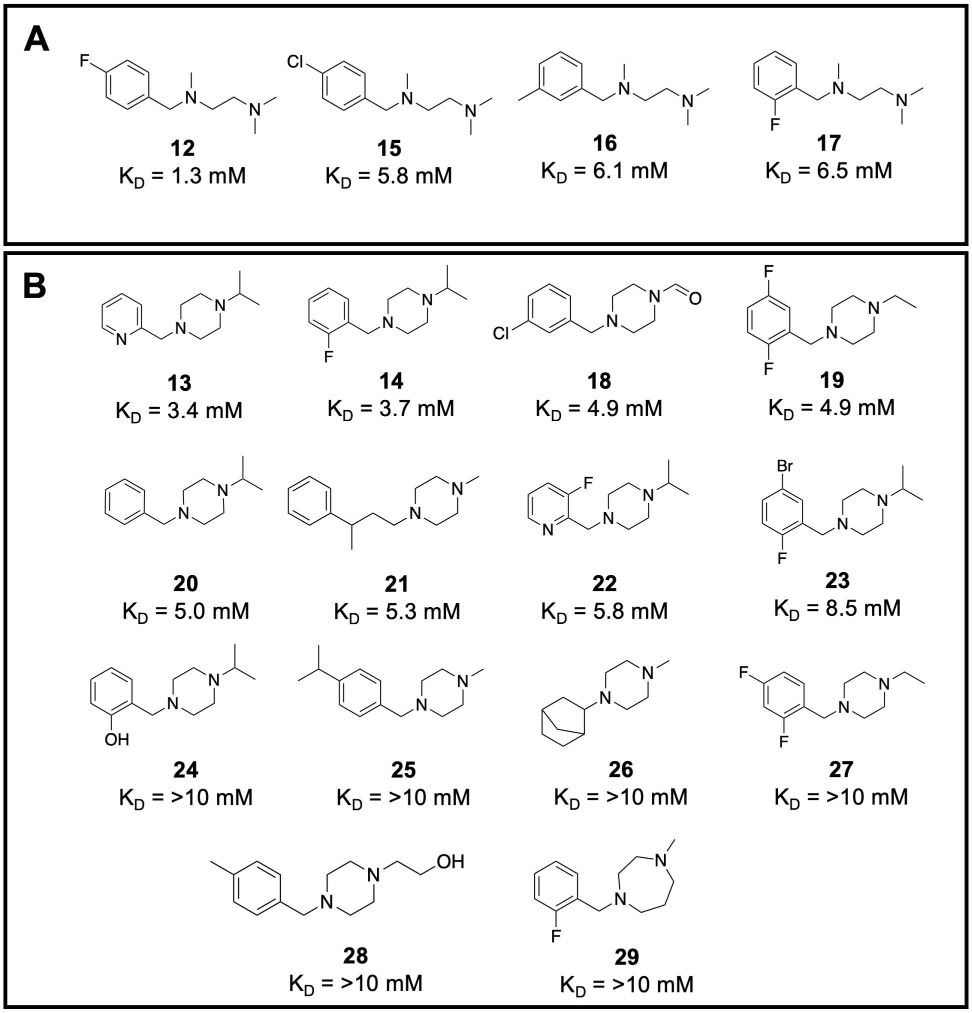

Figure S4. Chemical structures and K_D values of the Set 3 fragment hits of (A) benzyl diamines and (B) benzyl piperazines.

Table S1. X-ray data collection and refinement statistics of the VCB complex bound to fragments.

Compound	**9**	**13**	**14**	**15**
PDB Accession code	9OIM	9OIN	9OIO	9OIQ
**Data collection**				
Space Group	$P\ 4_1\ 2\ 2$	$P\ 4_1\ 2_1\ 2$	$P\ 4_1\ 2\ 2$	$P\ 4_1\ 2_1\ 2$
Cell Dimensions				
a, b, c (Å)	93.39, 93.39, 362.71	94.04, 94.04, 363.68	92.90, 92.90, 359.38	94.28, 94.28, 363.76
α, β, γ (°)	90.00, 90.00, 90.00	90.00, 90.00, 90.00	90.00, 90.00, 90.00	90.00, 90.00, 90.00
Resolution (Å)	48.83-2.61 (2.70-2.61)	49.08-2.41 (2.47-2.41)	48.49-2.30 (2.33-2.30)	49.15-2.66 (2.75-2.66)
R_{merge} (%)	0.072 (1.010)	0.064 (1.516)	0.1191 (2.022)	0.037 (0.277)
Mean I / σI	10.6 (0.7)	11.2 (0.7)	19.1 (2.0)	18.9 (2.8)
Completeness (%)	95.8 (97.0)	100 (100)	99.9 (99.9)	100 (100)
Redundancy	1.9 (1.9)	1.9 (1.9)	26.5 (28.0)	1.9 (1.9)
**Structure Refinement**				
No. Reflections	47,319	64,119	71,176	48,203
R_{work}/R_{free}	0.2250/0.2833	0.2328/0.2827	0.2132/0.2493	0.2263/0.2745
R.m.s. deviations				
Bond lengths (%)	0.010	0.009	0.009	0.009
Bond angles (%)	1.223	1.033	1.112	1.161
Ramachandran				
Preferred regions (%)	96.77	95.76	96.79	96.80
Allowed regions (%)	3.15	4.16	3.13	3.04
Disallowed regions (%)	0.08	0.08	0.08	0.16

*High resolution shells are in parentheses.

Experimental Procedures

Protein expression and purification

All gene synthesis, cloning, and sequencing in this work were conducted by GenScript. VHL gene (residues 54-213) was cloned into pET28a(+) vector. Elongin C (residues 17-112) and Elongin B (residues 1-104) genes were cloned into pCDFDuet-1. All plasmids were transformed to *E. Coli*. BL21(DE3) and grown on selective agar media supplemented with kanamycin and streptomycin. Bacteria were cultured at 37 ^{o}C in LB broth or M9 minimal media supplemented with $^{15}NH_4Cl$ for regular and isotope-labeled protein, respectively, until optical density at 600nm reaches 0.6. VCB co-expression was induced by 0.5mM isopropyl β-d-1-thiogalactopyranoside followed by further incubation at 25°C overnight. Cell pellet was harvested by centrifugation at 5,000g for 15 minutes and resuspended in lysis buffer (50mM HEPES pH 7.5, 300mM NaCl, 20mM imidazole, 5mM BME) and lysed using APV2000 Lab homogenizer (SPX Flow) at 600 bar. Cell lysate was centrifuged at 13,000g at 4°C for 1 hour to remove cell debris. Supernatant was loaded to HisTrap NiNTA column (Cytiva) pre-equilibrated with lysis buffer. The column was washed with 10xcolumn volume of lysis buffer and then eluted with 500mM imidazole in the same buffer using a linear gradient from 0-100% over 10xcolumn volume. The VCB complex was cleaved His-tag using thrombin and dialyzed to remove imidazole simultaneously at 4°C overnight. Tag-free VCB was concentrated and loaded to HiLoad 26/600 Superdex75 pg (Cytiva) and eluted with 20mM HEPES pH 8.0, 200mM NaCl, 1mM DTT for crystallography or 25mM sodium phosphate pH 7.5, 2mM DTT for NMR experiments. VCB concentration was determined by Pierce 660nm assay reagent (Thermofisher) using standard bovine albumin serum for making standard curve (Thermofisher).

NMR Experiments

All NMR experiments were carried out at 310K using a 900 MHz Bruker Avance III spectrometer equipped with a TXI/TCI CryoProbe and a Bruker SampleJet. Two-dimensional ^{1}H-^{15}N TROSY spectra of VCB complex were recorded using 30-minute acquisition time and analyzed using Topspin 4.1.4 (Bruker). Our in-house fragment library of 13,824 compounds was screened as mixtures of 12 fragments prepared in twelve 96-well plates. Each NMR sample was made of 100μM of ^{15}N-labeled VCB, 800μM of each fragment, and 5% DMSO-d_6 for spectrometer locking in 3mm-diameter NMR tubes (Bruker). Hit mixtures were identified by comparing the chemical shifts of the backbone resonances to a ligand-free VCB spectrum and then deconvoluted by screening individual fragments.

TROSY titration experiments were used to determine binding affinity of the fragment hits identified from the screen. The changes in ^{1}H-^{15}N chemical shifts of backbone resonances upon the addition of increasing concentrations of the fragments (0.3125-10 mM) were analyzed. The binding affinities (K_Ds) of the fragments were calculated using the Hill's equation model in Prism 10 (GraphPad).

Crystallography

VCB complex was concentrated to 7 mg/mL in crystallization buffer containing 20 mM Tris pH 7, 150 mM NaCl, and 1 mM DTT. VCB crystals were formed by hanging drop vapor diffusion against reservoir containing 0.1 M sodium cacodylate pH 5.5, 0.2 M magnesium acetate, 5 mM DTT and 14-20% PEG8000 at 291 K. VCB crystals were formed in drops with 1:1 or 2:1 ratio of protein:reservoir and were cryo-protected in 10% glycerol in reservoir solution. VCB in complex with fragments were generated by soaking ligand-free VCB crystals

for 24 hours at 291 K in crystallization conditions containing 100 mM of the fragment. VCB in complex with multiple molecules were generated by performing serial soaks with the first 24-hour soak containing one molecule and the second soak containing both fragments of interest at 100 mM concentration. X-ray diffraction experiments were performed at 100K on beamline 8.2.2 at the Advanced Light Source (ALS), Lawrence Berkely National Laboratory, California, USA. Diffraction data were processed using XDS.[1] Phasing was accomplished by molecular replacement with Phaser[2] using the structure of apo VCB complex (PDB:1VCB) as starting model. Ligand models were built by AceDRG[3] and manually added to the corresponding electron density. VCB co-crystal structures were determined by several cycles of refinement using Phenix[4] and manual modelling with COOT.[5]

References

(1) Kabsch, W. XDS. *Acta Crystallogr D Biol Crystallogr* 2010, *66* (2), 125–132. https://doi.org/10.1107/S0907444909047337.

(2) McCoy, A. J.; Grosse-Kunstleve, R. W.; Adams, P. D.; Winn, M. D.; Storoni, L. C.; Read, R. J. Phaser Crystallographic Software. *J Appl Crystallogr* 2007, *40* (4), 658–674. https://doi.org/10.1107/S0021889807021206.

(3) Long, F.; Nicholls, R. A.; Emsley, P.; Gražulis, S.; Merkys, A.; Vaitkus, A.; Murshudov, G. N. AceDRG: A Stereochemical Description Generator for Ligands. *Acta Crystallogr D Struct Biol* 2017, *73* (2), 112–122. https://doi.org/10.1107/S2059798317000067.

(4) Adams, P. D.; Pavel, V.; Chen, V. B.; Ian, W.; Echols, N.; Moriarty, N. W.; Read, R. J.; Richardson, D. C.; Jane, S.; Thomas, C. PHENIX : A Comprehensive Python-Based System for Macromolecular Structure Solution Research Papers. *Acta Crystallographica Section D* 2010, *66*, 213–221. https://doi.org/10.1107/S0907444909052925.

(5) Emsley, P.; Cowtan, K. Coot: Model-Building Tools for Molecular Graphics. *Acta Crystallogr D Biol Crystallogr* 2004, *60* (12), 2126–2132. https://doi.org/10.1107/S0907444904019158.
